# FOXO3a-dependent PARKIN negatively regulates cardiac hypertrophy by restoring mitophagy

**DOI:** 10.1186/s13578-022-00935-y

**Published:** 2022-12-19

**Authors:** Teng Sun, Yu Han, Jia-Lei Li, Xiang-Ying Jiao, Lin Zuo, Jin Wang, Hai-Xiong Wang, Jun-Li Yang, Ji-Min Cao, Jian-Xun Wang

**Affiliations:** 1 Key Laboratory of Cellular Physiology at Shanxi Medical University, Ministry of Education, Key Laboratory of Cellular Physiology of Shanxi Province, and the Department of Physiology, Shanxi Medical University, Taiyuan, China; 2 Department of Cardiology, Shanxi Cardiovascular Hospital, Taiyuan, Shanxi China; 3 Computer teaching department, Shanxi Medical University, Taiyuan, China; 4 School of Basic Medical Sciences, Qingdao University, Qingdao, China

**Keywords:** PARKIN, FOXO3a, Mitophagy, Cardiac hypertrophy

## Abstract

**Background:**

Sustained cardiac hypertrophy often develops maladaptive myocardial remodeling, and eventually progresses to heart failure and sudden death. Therefore, maladaptive hypertrophy is considered as a critical therapeutic target for many heart diseases. Mitophagy, a crucial mechanism in mitochondria quality control and cellular homeostasis, has been implicated in diverse cardiac disorders such as myocardial infarction, diabetic cardiomyopathy, cardiac hypertrophy and heart failure. However, what role mitophagy plays in heart diseases remains an enigma. PARKIN functions as an E3 ubiquitin protein ligase and mediates mitophagy cascades. It is still unclear whether PARKIN participates in the regulation of cardiac hypertrophy.

**Results:**

PARKIN was downregulated in cardiomyocytes and hearts under hypertrophic stress. Enforced expression of PARKIN inhibited Ang II-induced cardiomyocyte hypertrophy. Compared to wide-type mice with Ang II-induced cardiac hypertrophy, *Parkin* transgenic mice subjected to Ang II administration showed attenuated cardiac hypertrophy and improved cardiac function. In addition, mitophagy machinery was impaired in response to Ang II, which was rescued by overexpression of PARKIN. PARKIN exerted the anti-hypertrophy effect through restoring mitophagy. In further exploring the underlying mechanisms, we found that PARKIN was transcriptionally activated by FOXO3a. FOXO3a promoted mitophagy and suppressed cardiac hypertrophy by targeting *Parkin*.

**Conclusions:**

The present study reveals a novel cardiac hypertrophy regulating model composed of FOXO3a, PARKIN and mitophagy program. Modulation of their levels may provide a new approach for preventing cardiac hypertrophy and heart failure.

**Graphical Abstract:**

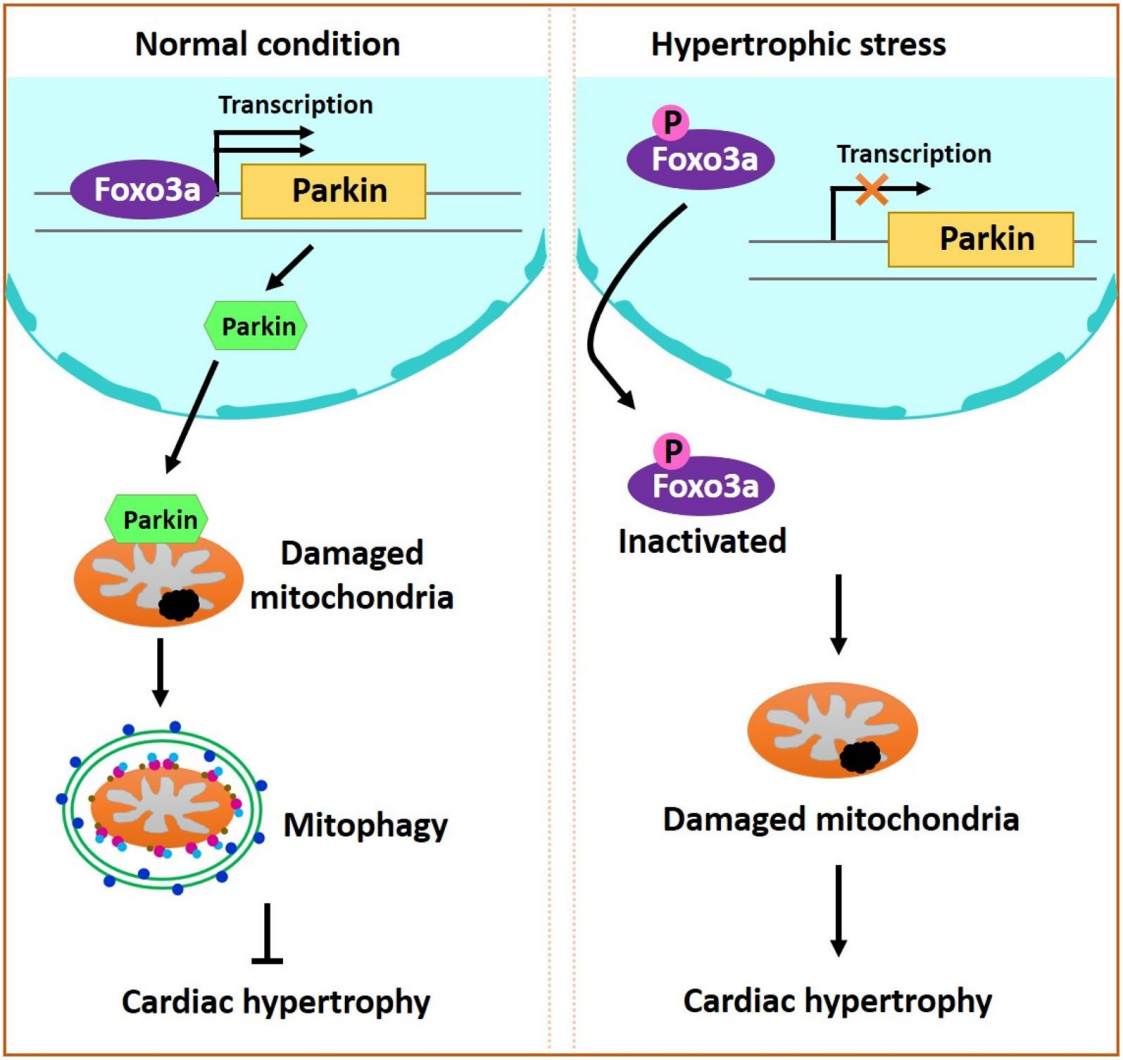

**Supplementary Information:**

The online version contains supplementary material available at 10.1186/s13578-022-00935-y.

## Background

Cardiac hypertrophy is a compensatory response to cardiac stress such as development, repetitive endurance exercise, pressure overload, volume overload, hypoxia, storage diseases, and inherited diseases. However, sustained cardiac hypertrophy usually progresses to maladaptive cardiac remodeling involving fibrosis, cardiomyocyte death, increased collagen synthesis, decreased pumping ability, arrhythmias, and aberrant gene expression, eventually leading to heart failure and even sudden cardiac death [1]. Therefore, pathological cardiac hypertrophy is considered as a critical therapeutic target for many heart diseases. Hitherto, hypertrophic signaling pathways, involving calcineurin/nuclear factor of activated T cell (NFAT) [2], AMP-activated protein kinase (AKT)/mammalian target of rapamycin (mTOR) [3], Ca^2^+/calmodulin-dependent protein kinase (CaMK)II^4^, cyclic guanosine monophosphate (cGMP)-protein kinase G (PKG) [5], mitogen-activated protein kinase (MAPK) [6], and non-coding RNAs [7], have been identified. Nevertheless, the fundamental mechanisms underlying pathological cardiac hypertrophy, especially the involvement of mitophagy program, are still poorly understood.

Mitophagy, a selective autophagic response, specifically the traffics of superfluous, aging or damaged mitochondria to lysosomes for degradation, serves as a crucial mechanism in mitochondrial quality control and cellular homeostasis. Depending on mitophagy, paternal mitochondria are eliminated from the fertilized eggs [8] and mitochondria are cleaned progressively during erythrocytes maturation [9]. Since mitochondria are the major energy production organelle in cardiomyocytes, mitophagy is particularly necessary to heart development and maintenance of cardiac homeostasis. Mitophagy defects contribute greatly to cardiac dysplasia and cardiac aging [10, 11]. Recent advance has revealed that mitophagy is implicated in the pathogenesis of heart diseases. It has been demonstrated that mitophagy is repressed in myocardial infarction and heart failure, and mitophagy reversion using genetical or pharmacological methods attenuates myocardial injury and remodeling, and improves cardiac function [12–14]. Diabetic cardiomyopathy is exacerbated by impaired mitophagy and prevented by activated mitophagy [15]. Conversely, other studies revealed that advanced heart failure and pathologic hypertrophy are accompanied by dramatically increased mitophagy. Inhibition of mitophagy rescues cardiac remodeling and heart failure [16, 17]. Given all above, what a role mitophagy plays in heart diseases, either positive or negative, and how it plays, are still enigmas.

PARKIN, also known as PARK2, is an E3 ubiquitin ligase and participates in multiple cellular processes and mitochondrial homeostasis through modulating post-translational modification of proteins in a ubiquitin–proteasome dependent pathway. By ubiquitinating receptor-interacting serine/threonine-protein kinase 3 (RIPK3) and Cyclophilin-D (CYPD), PARKIN prevents both receptor-dependent and mitochondrial ways of necroptosis [18, 19]. Defects on mono-ubiquitination of VDAC1 by PARKIN promotes apoptosis by augmenting the mitochondrial calcium uptake [20]. Loss of *Parkin* impairs mitochondrial biogenesis and mitochondrial respiration, leading to cell death [21]. Another widely appreciated role of PARKIN is mediating mitophagy. Upon phosphorylation by mitochondrial outer membrane (MOM)-localized PTEN-induced putative kinase protein-1 (PINK1), PARKIN is recruited to MOM and extensively catalyzes poly-ubiquitination of a dozen OMM substrates among which modified mitofusion (MFN) 1/2, MIRO and VDAC1 have been demonstrated to recruit autophagy adaptor such as p62/sequestosome 1 (SQSTM1), NDP52, and optineurin (OPTN), and ultimately target mitochondria removal [22–26]. Affluent effectors downstream of PARKIN-mediated mitophagy have been identified. However, the upstream triggering mechanism remains to be elucidated fully.

Albeit *Parkin* was first identified in Parkinson’s disease (PD), emerging evidence indicates that *Parkin* is deeply implicated in cardiovascular system. During heart development, PARKIN evokes the switch of mitochondria from nascent to mature through mediating fetal mitochondria degradation. Cardiomyocyte-specific deletion of *Parkin* at birth results in perinatal cardiomyopathy and premature death [27]. PARKIN is required for melatonin-mediated inhibition of mitochondrial dysfunction and cardiac remodeling in diabetic cardiomyopathy [28]. Our previous work has demonstrated that PARKIN alleviates cardiac ischemia/reperfusion injury and improved cardiac function [19]. The *Parkin*-deficient mice exhibited aggravated cardiac injury and increased mortality in response to myocardial infarction [29]. PARKIN participates in cardiac physiological and pathological processes, but the underlying mechanism, especially referring to hypertrophic program, is largely unknown. Thus, we were interested in exploring the role of PARKIN in cardiac hypertrophy.

Forkhead box O3a (FOXO3a), a member of forkhead family of transcription factors, extensively regulates gene transcription depending on its 100-amino acid DNA binding domain. In a state of non-phosphorylation and deacetylation, FOXO3a regulates diverse cellular functions, including proliferation, differentiation, metabolism, cell death, and stress response [30–33]. FOXO3a is highly expressed in hearts and functions as a negative regulator in cardiac disorders. FOXO3a inhibits cardiac apoptotic and necrotic cell death, and maintains calcium homeostasis in response to myocardial infarction. *Foxo3a* transgenic mice exhibit reduced cell death and infarct size, and improved cardiac function [30, 31, 34]. Phosphorylated FOXO3a accumulates upon hypertrophic stimuli such as insulin, angiotensin II (Ang II), phenylephrine, and pressure overload, and enforced expression of FOXO3a inhibits cardiac hypertrophy [35, 36]. FOXO3a suppresses mitochondrial fission and apoptosis, and protects against doxorubicin-induced cardiotoxicity [32]. Recent advances have shown that FOXO3a is implicated in mitophagy signaling pathways. FOXO3a can upregulate the expression level of Bcl-2 E1B 19-KDa interacting protein 3 (BNIP3), an autophagy receptor partially mediating non-canonical mitophagy [17]. Loss of *Sirt3* impairs autophagy and mitophagy accompanied by decreased deacetylation of FOXO3a and levels of PARKIN [37]. FOXO3a is seemingly co-localized with mitochondrial PINK1/PARKIN proteins activated by antioxidants [38]. However, the role of FOXO3a in mitophagy and whether FOXO3a targets mitophagy program in the pathogenesis of cardiac disorders is still poorly understood.

The present study aimed at exploring the role of PARKIN in cardiac hypertrophy. Mitophagy is impaired upon hypertrophic stimulation. PARKIN was found to regulate cardiac hypertrophy by modulating mitophagy process. *Parkin* transgenic mice exhibits rescued mitophagy, decreased hypertrophic responses, and improved cardiac function. In searching for the upstream regulators of PARKIN, we identified that FOXO3a transcriptionally activated PARKIN expression. FOXO3a participated in regulating mitophagy and cardiac hypertrophy through targeting PARKIN. Taken together, our results revealed a novel hypertrophic regulating model composed of FOXO3a, PARKIN and mitophagy program.

## Results

### PARKIN suppresses cardiac hypertrophy in the heart

**Fig. 1:**
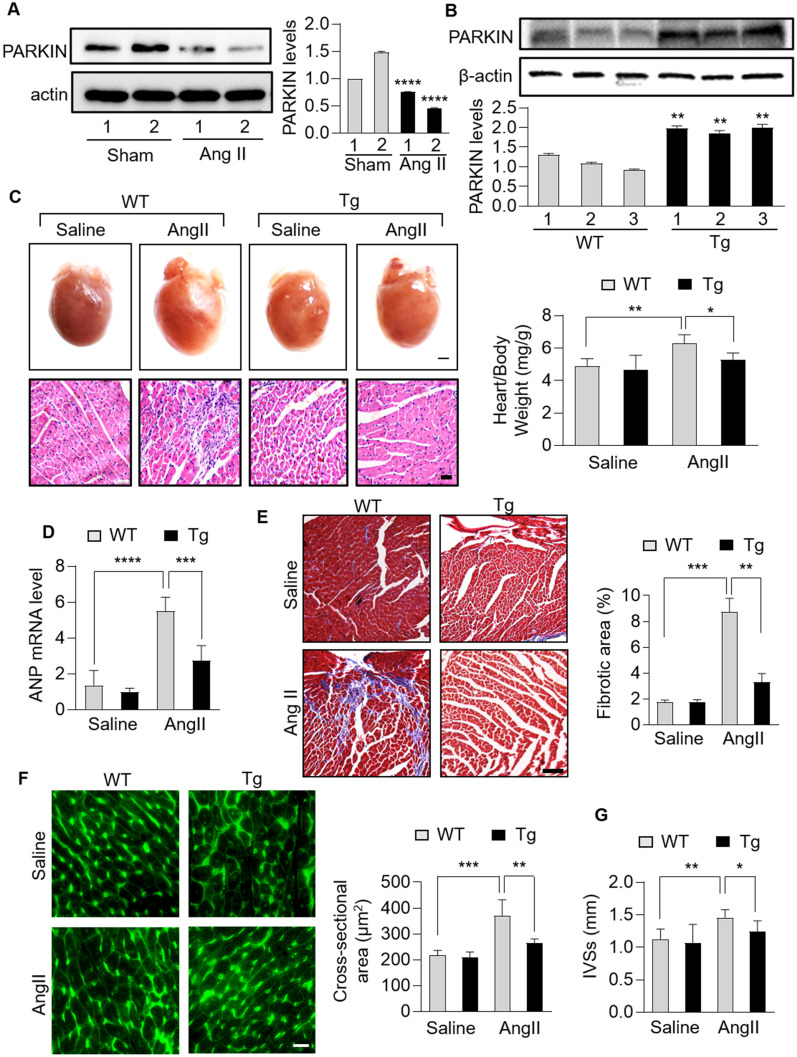
PARKIN negatively regulated cardiac hypertrophy in mice. **A**, Immunoblotting results showing the protein levels of PARKIN in mice hearts infused with angiotensin (Ang II) or not. n = 3 experiments per group. **B**, Immunoblotting results showing the protein levels of PARKIN in hearts of *Parkin* transgenic mice or wide-type (WT) mice. n = 3 experiments per group. **C − E**, *Parkin* transgenic mice exhibited reduced hypertrophic responses and cardiac fibrosis. *Parkin* transgenic mice and WT mice were infused with Ang II. **C** Top row: gross hearts (bar = 1.5 mm); bottom row: heart sections stained with hematoxylin and eosin (bar = 20 µm); right: the ratio of heart weight to body weight. * *p* < 0.05. ** *p* < 0.01. n = 5 experiments per group. **D** The mRNA levels of atrial natriuretic peptide (ANP) and brain natriuretic peptide (BNP) detected by qRT-PCR. The results were normalized to GAPDH. *** *p* < 0.001. ****p < 0.0001. n = 6 experiments per group. (E) Heart sections stained with Masson trichrome (left) and the fibrotic area analysis (right) (bar = 50 µm); ** *p* < 0.01. *** *p* < 0.001. n = 5 experiments per group. **F** and **G**, *Parkin* transgenic mice exhibited reduced cardiac remodeling and improved cardiac function in response to Ang II. **F** TRITC-conjugated wheat germ agglutinin staining was used to assessed cross-sectional area of hearts from WT mice and *Parkin* transgenic mice with Ang II infusions (left). The cross-sectional area was calculated (right). Bar = 100 µm; ** *p* < 0.01. *** *p* < 0.001. n = 6 experiments per group. **G** End-systolic interventricular septum thickness (IVSs) was measured. * *p* < 0.05. ** *p* < 0.01. n = 6 experiments per group

There is evidence that PARKIN is highly expressed in the heart and participates in heart development and pathogenesis of cardiac disorders, but the underlying mechanism, especially referring to cardiac hypertrophy, is largely unknown. Ang II has been well demonstrated to induce cardiac hypertrophy [19, 27–29]. In Ang II-induced hypertrophic hearts, PARKIN was downregulated significantly (Fig. 1A). To further investigate the role of PARKIN in cardiac hypertrophy, we constructed cardiac-specific *Parkin* transgenic mice using a myosin heavy-chain (MHC) promoter. The level of PARKIN in hearts of *Parkin*-transgenic mice was much higher than that in wide-type mice (Fig. 1B). Increased heart weight to body weight ratio and increased mRNA level of atrial natriuretic peptide (ANP) were observed in wide-type mice administrated with Ang II, while these hypertrophy responses were significantly reduced in *Parkin* transgenic mice (Fig. 1C, D). In addition, *Parkin* transgenic mice showed a suppressed interstitial fibrosis, as assessed by hematoxylin–eosin staining and Masson trichrome staining (Fig. 1C, E). Next, cardiac function in animal models were measured. Compared with that in wide-type mice, attenuated cardiac remodeling and improved heart function were exhibited in *Parkin* transgenic mice (Fig. 1F, G).

## PARKIN inhibits Ang II-induced cardiomyocyte hypertrophy

**Fig. 2:**
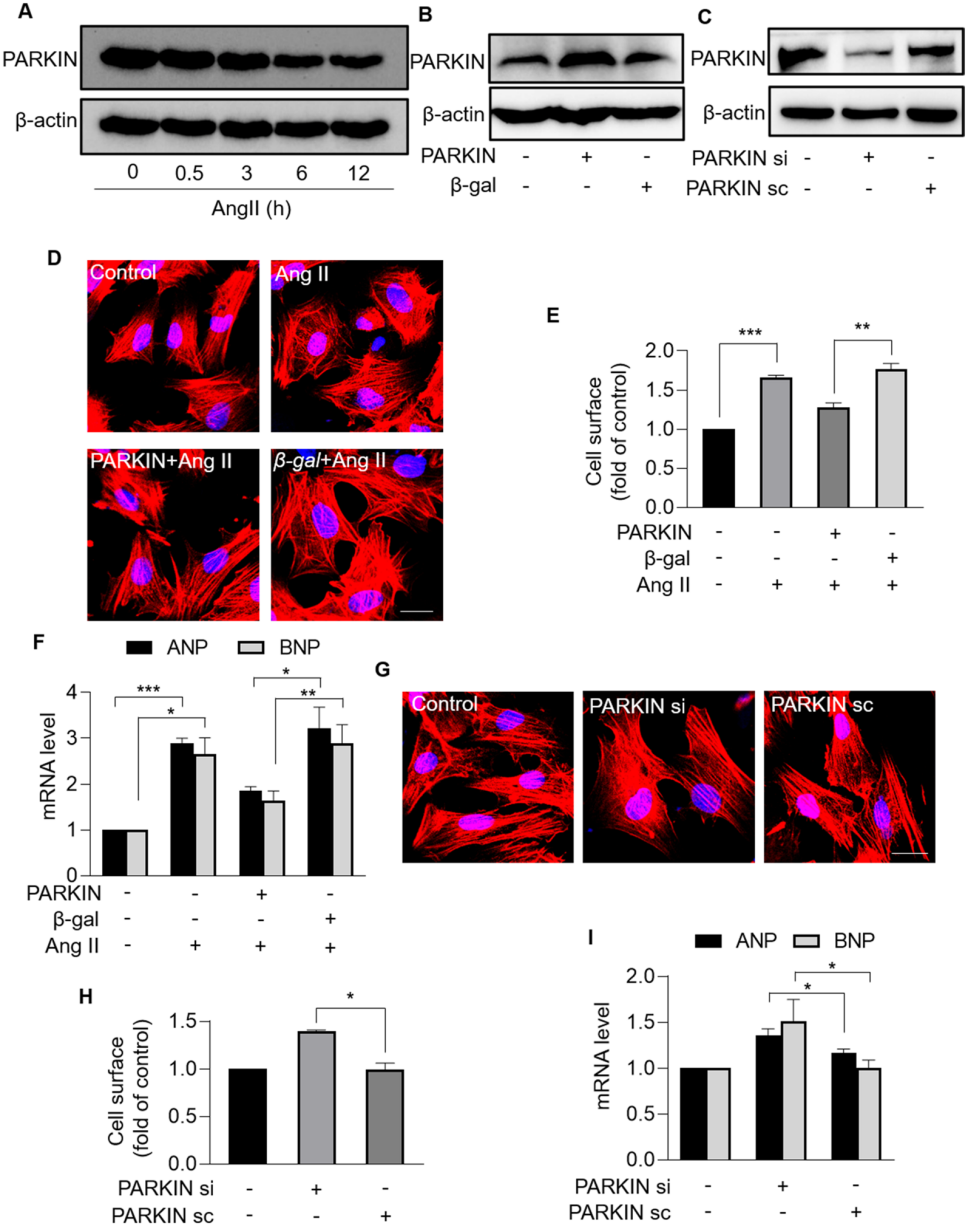
PARKIN inhibited Ang II-induced cardiomyocyte hypertrophy. **A**, Immunoblotting results exhibiting the protein levels of PARKIN in cardiomyocytes treated with Ang II at the indicated time. **B**, Protein levels of PARKIN in cardiomyocytes infected with *Parkin* adenovirus or β-gal adenovirus. **C,** Protein levels of PARKIN in cardiomyocytes infected with PARKIN siRNA or PARKIN scramble. **D − F**, Overexpression of PARKIN suppressed Ang II-induced cardiomyocyte hypertrophy. Cardiomyocytes infected with *Parkin* adenovirus or β-gal adenovirus were exposed to Ang II. (D) Sarcomere organization stained with phalloidin-TRITC conjugate; bar = 20 µm. Blue represent nucleus. Red represent F-actin. (E) Cell surface was calculated. ** *p* < 0.01. *** *p* < 0.001. n = 3 experiments per group. (F) The mRNA levels of ANP and BNP were analyzed by qRT-PCR. The results were normalized to GAPDH. * *p* < 0.05. **p < 0.01. *** *p* < 0.001. n = 3 experiments per group. **G** − **I**, Knockdown of PARKIN induced hypertrophic responses. Cardiomyocytes were infected with PARKIN siRNA or PARKIN scramble. **G** Sarcomere organization stained with Phalloidin-TRITC conjugate; bar = 20 µm. Blue represent nucleus. Red represent F-actin. **H** Cell surface was calculated. * *p* < 0.05. n = 3 experiments per group. **I** The mRNA levels of ANP and BNP were analyzed by qRT-PCR. The results were normalized to GAPDH. * *p* < 0.05. n = 3 experiments per group

We further studied the role of PARKIN in cardiac hypertrophy at the cellular level. The expression level of PARKIN was detected in neonatal rat cardiomyocytes treated with Ang II. PARKIN was downregulated in response to Ang II in a time-dependent manner (Fig. 2A). To investigate whether PARKIN regulated cardiac hypertrophy, adenovirus expressing dominant PARKIN or PARKIN siRNAs was used to modulate PARKIN levels (Fig. 2B, C). Enforced expression of PARKIN significantly attenuated Ang II-induced hypertrophy, including reduced sarcomere organization (Fig. 2D), decreased cell surface (Fig. 2E), and decreased levels of hypertrophic marker atrial natriuretic peptide (ANP) and brain natriuretic peptide (BNP) (Fig. 2F). Moreover, *Parkin*-deficient cardiomyocytes exhibited hypertrophic responses in the absence of Ang II, as evidenced by increased sarcomere organization (Fig. 2G), increased cell surface (Fig. 2H), and increased ANP and BNP levels (Fig. 2I). Taken together, PARKIN inhibits Ang II-induced hypertrophy in cardiomyocytes.

## Mitophagy is damaged under hypertrophic stress

**Fig. 3:**
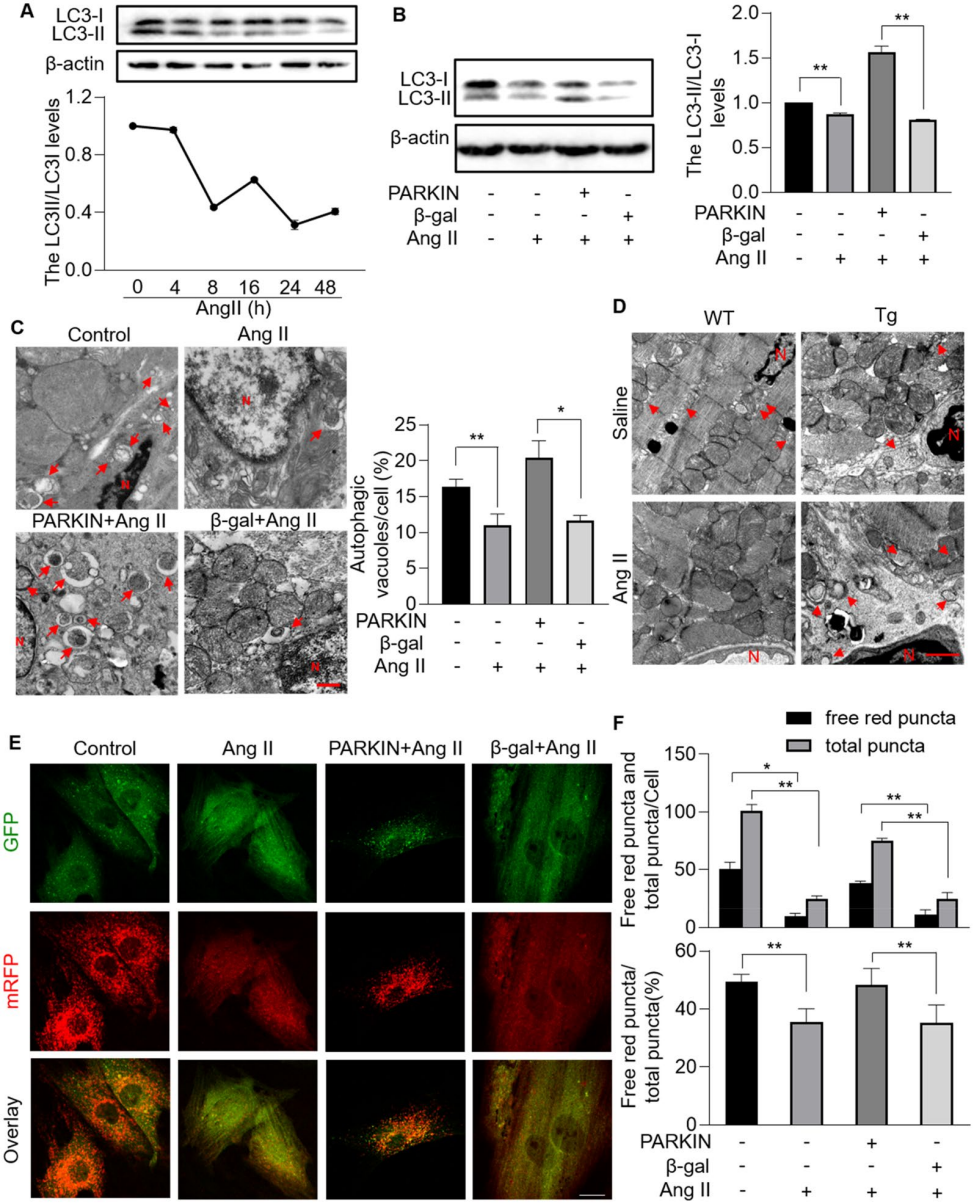
Mitophagy was impaired in Ang II-treated cardiomyocytes and hearts, which was rescued by overexpression of PARKIN. **A**, Immunoblotting results showing the protein levels of light chain 3 (LC3) I and LC3 II in cardiomyocytes exposed to Ang II at the indicated time. n = 3 experiments per group. The ratio of LC3II/LC3I was calculated. **B**, Enforced expression of PARKIN rescued Ang II-induced decreased LC3II/LC3I ratio. Cardiomyocytes infected with *Parkin* adenovirus or β-gal adenovirus were exposed to Ang II. Immunoblot was performed to detect protein levels of LC3I and LC3II (left). n = 3 experiments per group. The ratio of LC3II/LC3I was calculated (right). ** *p* < 0.01. **C** and **D**, PARKIN restored autophagic vacuoles in hypertrophic model. Autophagic vacuoles were visualized in cardiomyocytes infected with *Parkin* adenovirus or β-gal (C, left; bar = 500 nm), and hearts of *Parkin* transgenic mice or WT mice (D; bar = 1 µm). Quantitation of autophagic vacuoles were shown in (C, right). * *p* < 0.05. ** *p* < 0.01. n = 3 experiments per group. **E** and **F**, Overexpression of PARKIN attenuated Ang II-induced mitophagy flux defects. LC3 adenovirus tandem-labeled green fluorescent protein (GFP)-monomeric red fluorescent protein (mRFP) (GFP-mRFP-LC3) was used to indicate mitophagy flux. GFP-mRFP-LC3 was expressed and detected in 24 h after transfection in cardiomyocytes with overexpression or PARKIN or not. GFP-LC3 (green puncta), mRFP (red puncta, representative of autolysosomes) and overlay (yellow puncta, representative of autophagosomes) (**E**; bar = 25 µm). Quantification results were shown in (**F**). * *p* < 0.05. ** *p* < 0.01. n = 3 experiments per group

Mitophagy is an evolutionarily conserved lysosome-dependent mitochondrial degradation mechanism and contributes greatly to mitochondrial quality control. Mitophagy defects, deficiency or excess lead to cellular dysfunction and eventually heart diseases [8, 10, 13]. However, the role of mitophagy in the pathogenesis of cardiac dysfunction remains elusive. To explore whether mitophagy is implicated in cardiac hypertrophy, we evaluated mitophagy levels in Ang II-treated cardiomyocytes. Light chain 3 (LC3) protein sequentially undergoes a series of modification including removing carboxyl terminal region (form LC3-I), and conjugating to phosphatidylethanolamine (form LC3-II), and finally binding to the membrane of autophagosomes during autophagy process. LC3 is therefore widely used as a reliable autophagosome marker and the lipidated LC3-II monitors the occurrence of autophagosome formation [39]. We observed that the ratio of LC3-II/LC3-I was decreased upon Ang II treatment in a time-dependent manner (Fig. 3A, B). The accumulation of LC3II puncta provides an effective way to detect autophagosomes. Reduced GFP-LC3II puncta co-localized with mitochondria were observed in cardiomyocytes exposed to Ang II (Additional file 1: Figure S1), which indicated that Ang II impaired mitophagy. To consolidate the altered mitophagy levels, transmission electron microscopy (TEM) was performed to observe the ultrastructure of mitochondria. Remarkably, mitophagic vacuoles accumulation enveloping damaged mitochondria was decreased both in cardiomyocytes and hearts in response to Ang II stimulation (Fig. 3C, D and Additional file 2: Fig. S2). Taken together, mitophagy is damaged under Ang II-induced hypertrophic stress.

Next, we dissected whether there was any defect in mitophagic flux in cardiomyocytes exposed to Ang II. LC3 adenovirus tandem-labeled green fluorescent protein (GFP)-monomeric red fluorescent protein (mRFP) (GFP-mRFP-LC3) was introduced. The green fluorescence was weakened when autophagosome fused to lysosome, as GFP was normally deprotonated in acidic lysosomes. Thus, yellow puncta indicated autophagosome and free red puncta indicated autolysosomes. Ang II attenuated the transformation of autophagosome to autolysosome, as indicated by decreased ratio of free red puncta/total puncta (Fig. 3E, F). In conclusion, mitophagy process was impaired and mitophagic flux was interdicted under hypertrophic stress.

### PARKIN blocks hypertrophic signal by restoring mitophagy

**Fig. 4:**
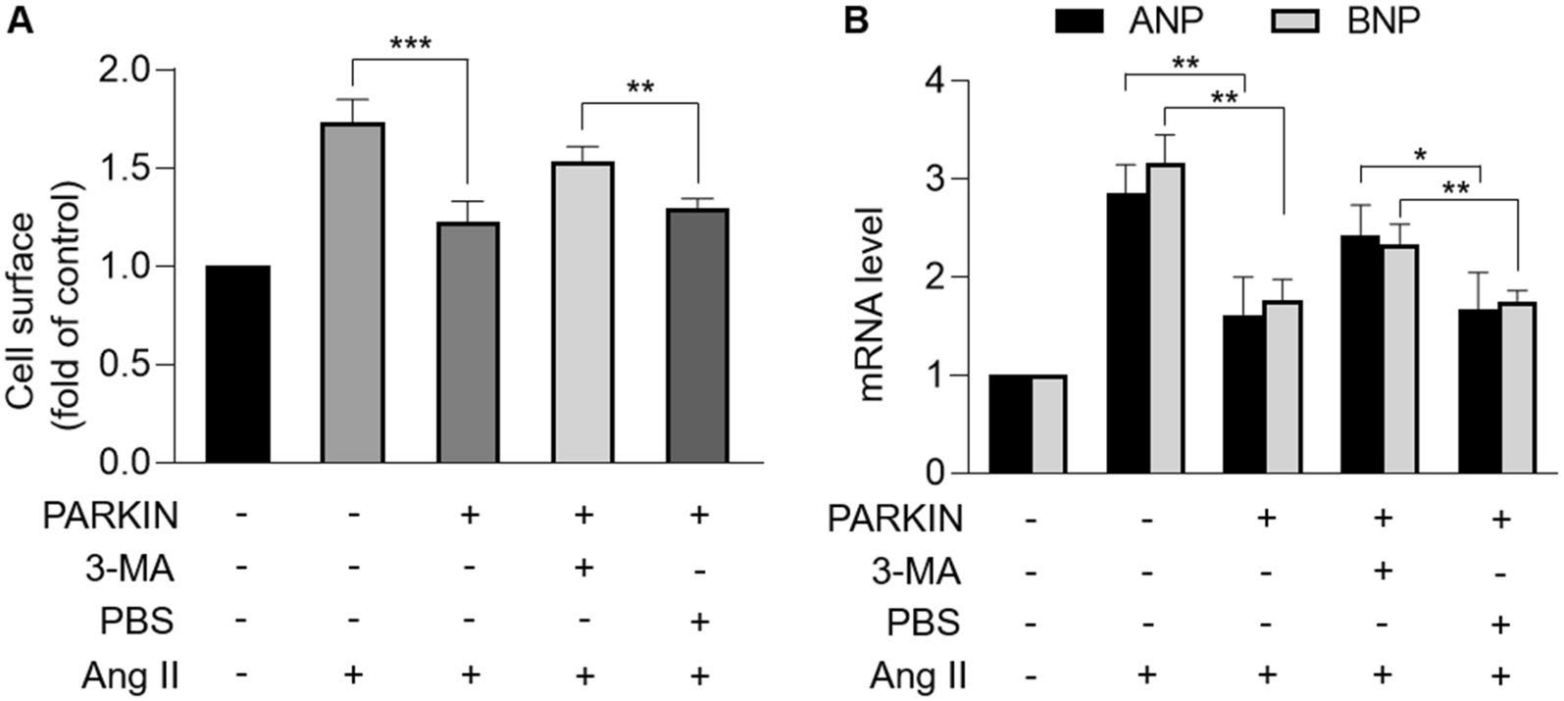
PARKIN regulated cardiac hypertrophy through targeting mitophagy. After being infected with *Parkin* adenovirus, cardiomyocytes were treated with 3-methyladenine (3-MA) or PBS, and then exposed to Ang II. **A**, Cell surface area indicated by phalloidin-TRITC conjugate stained F-actin were calculated. ** *p* < 0.01. *** *p* < 0.001. n = 3 experiments per group. **B**, mRNA levels of ANP and BNP. * *p* < 0.05. ** *p* < 0.01. n = 3 experiments per group

PARKIN is documented to convey mitophagic signals through catalyzing ubiquitination of outer mitochondrial membrane (OMM) substrates [26]. Therefore, we investigated whether PARKIN regulates mitophagy under hypertrophic stress. Results showed that Ang II-downregulated LC3-II/LC3-I ratio was reversed by enforced expression of PARKIN (Fig. 3B). More GFP-LC3II puncta co-localized with mitochondria were observed upon overexpression of PARKIN than that of negative control group (Additional file 1: Fig. S1). Increased mitophagic vacuoles enveloping damaged mitochondria were observed by TEM in cardiomyocytes overexpressing PARKIN and hearts of *Parkin* transgenic mice under hypertrophic stress (Fig. 3C, D and Additional file 2: Figure S2A). In addition, increased PARKIN attenuated the inhibition of mitophagic flux by Ang II, as evidenced by increased ratio of free red puncta/total puncta (Fig. 3E, F). These results indicated that PARKIN attenuated Ang II-induced mitophagy defects. Next, we dissected whether PARKIN exerted its anti-hypertrophy effect through activating mitophagy. The 3-methyladenine (3-MA), a class III phosphatidylinositol 3-kinase (PI3K) inhibitor, was used to inhibit autophagy process. Enforced expression of PARKIN attenuated Ang II-induced hypertrophic responses with significantly increased cell surface area and increased levels of ANP and BNP, which was abolished by 3-MA (Fig. 4A, B). Taken together, PARKIN negatively regulated cardiac hypertrophy through restoring mitophagy.

### PARKIN is transcriptionally activated by FOXO3a

**Fig. 5:**
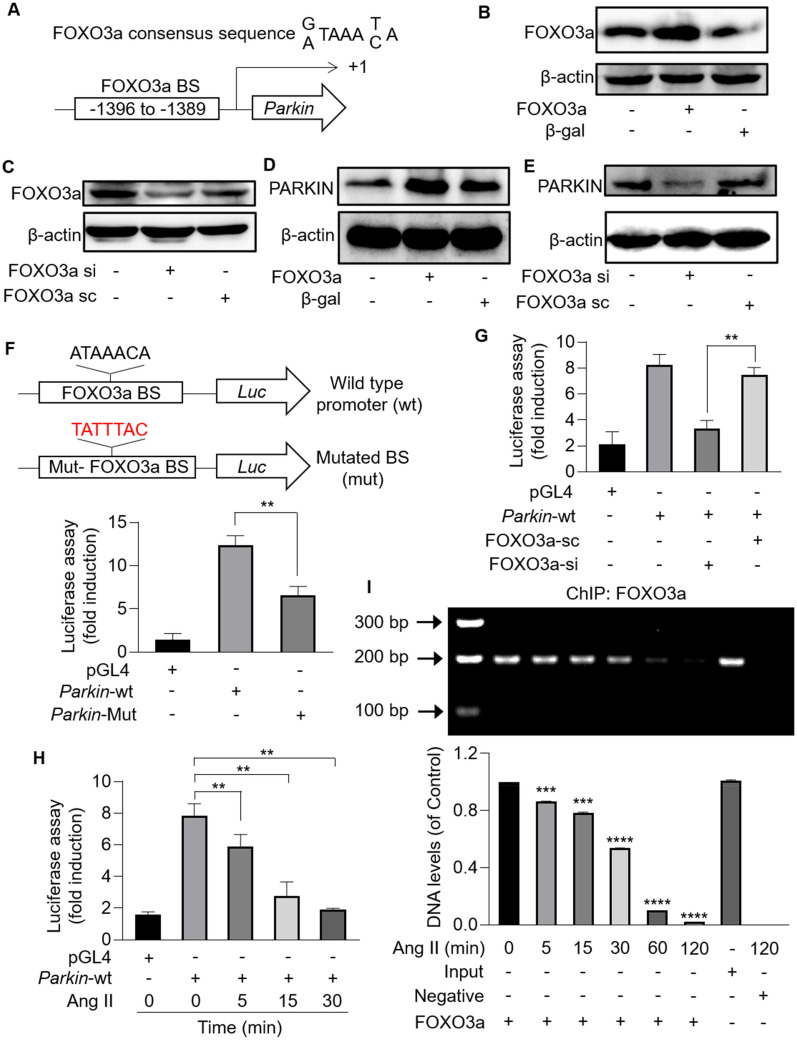
*Parkin* was a transcriptional target of FOXO3a. **A**, The promoter region of *Parkin* contains an optimal FOXO3a binding site. **B** and **C**, Immunoblotting results showing the protein levels of FOXO3a in cardiomyocytes infected with FOXO3a adenovirus or FOXO3a siRNA. n = 3 experiments per group. **D** and **E**, FOXO3a promoted PARKIN expression in cardiomyocytes. PARKIN increased upon overexpression of FOXO3a (D), while PARKIN decreased upon knockdown of FOXO3a (E). n = 3 experiments per group. **F**, *Parkin* promoter fragment containing FOXO3a potential binding site and its mutated fragment were cloned into luciferase reporter gene vector (pGL4.17), respectively (top). HEK293 cells were transfected with pGL4.17 vector expressing *Parkin* wide-type promoter or mutated promoter. The firefly luciferase activities were measured. ** *p* < 0.01 (bottom). n = 3 experiments per group. **G**, The firefly luciferase activities were detected in cardiomyocytes transfected with pGL4.17 vector expressing *Parkin* WT promoter and FOXO3a siRNA. ** *p* < 0.01. n = 3 experiments per group. **H** and **I**, Binding between FOXO3a and *Parkin* promoter was destroyed by Ang II. **H** Cardiomyocytes were exposed to Ang II, after transfected with pGL4.17 expressing *Parkin* WT promoter. The luciferase activities were attenuated upon Ang II treatment in a time-dependent manner. * *p* < 0.05. **I** Cardiomyocytes were treated with Ang II at the indicated time for ChIP analysis. Chromatin-bound DNA was immunoprecipitated with anti-FOXO3a antibody. Immunoprecipitated DNA was analyzed by PCR using a pair of primers combination that encompassed FOXO3a binding site. *** *p* < 0.001. **** *p* < 0.0001. n = 3 experiments per group

To investigate the regulatory mechanisms involved in PARKIN-mediated mitophagy and PARKIN-regulated hypertrophy, we analyzed the promoter region of *Parkin*. One putative consensus binding site for FOXO3a was identified, located at 1389 bp − 1396 bp upstream from the transcription starting site (Fig. 5A). Thus, adenovirus expressing FOXO3a or FOXO3a siRNA were used to modulate FOXO3a levels (Fig. 5B, C). Enforced expression of FOXO3a upregulated PARKIN levels, while knockdown of FOXO3a downregulated PARKIN levels (Fig. 5D, E). To clarify the interaction between FOXO3a and *Parkin*, dual-luciferase assay was performed. *Parkin* promoter sequence containing FOXO3a potential binding sites or mutated binding sites was cloned into pGL4.17 luciferase reporter gene vectors (Fig. 5F). HEK293 cells transfected with pGL4.17 vector expressing *Parkin* wide-type promoter showed substantially enhanced chemiluminescence signal than that transfected with pGL4.17 vector. However, the signal reduced upon mutation of FOXO3a potential binding sites (Fig. 5F) or knockdown of FOXO3a (Fig. 5G). These results indicated that FOXO3a activated *Parkin* transcription by directly binding to the consensus sequence located at the promoter region of *Parkin*. Next, we analyzed the interaction between FOXO3a and *Parkin* in cardiomyocytes under hypertrophic stress. In cardiomyocytes transfected with pGL4.17 vector expressing *Parkin* wide-type promoter, a time-dependent decrease of chemiluminescence signal was detected upon Ang II treatment (Fig. 5H). The chromatin immunoprecipitation (ChIP)-PCR analysis showed that abundant *Parkin* promoter fragments containing FOXO3a binding sites were detected in cardiomyocytes, which were attenuated in response to Ang II in a time-dependent manner (Fig. 5I). Taken together, FOXO3a transcriptionally upregulated PARKIN expression levels by directly binding to the promoter region of *Parkin*, which could be inhibited by Ang II.

### FOXO3a negatively regulates cardiac hypertrophy

**Fig. 6:**
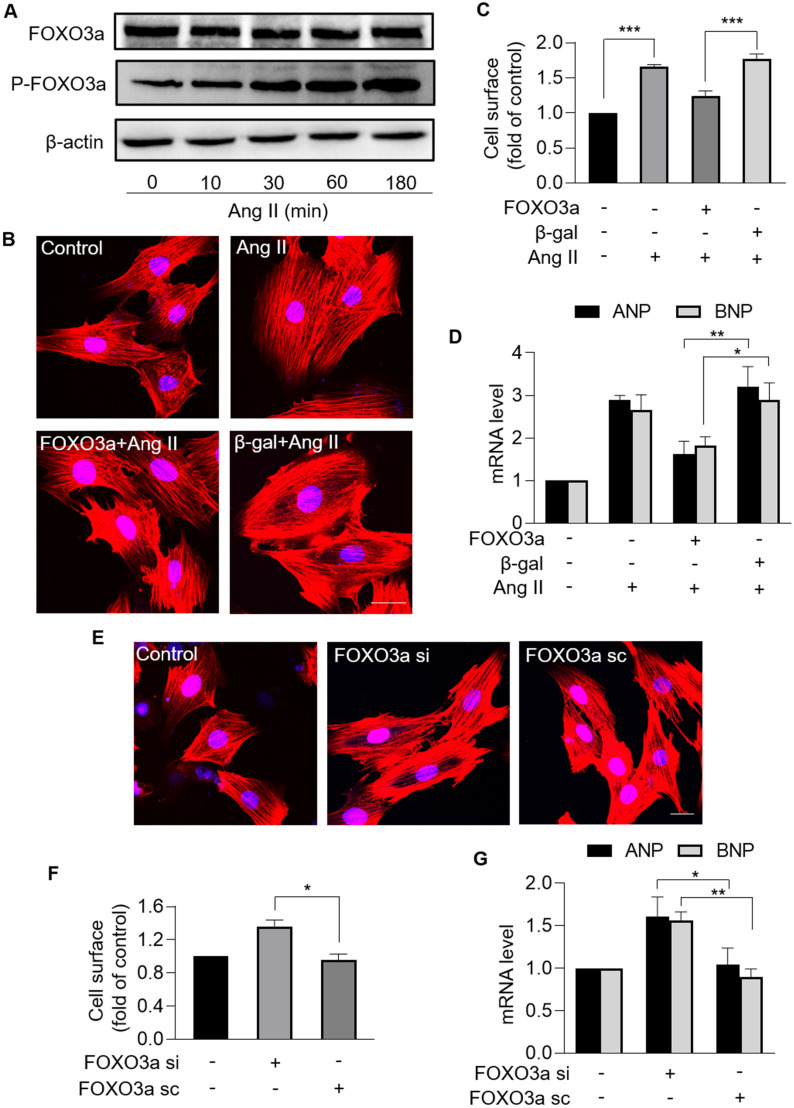
FOXO3a protected against cardiomyocyte hypertrophy. **A**, Immunoblotting results of the protein levels of total FOXO3a and phosphorylated FOXO3a in cardiomyocytes treated with Ang II at the indicated time. n = 3 experiments per group. **B** − **D**, Overexpression of FOXO3a inhibited Ang II-induced cardiomyocyte hypertrophy. Cardiomyocytes were exposed to Ang II after infected with FOXO3a adenovirus or β-gal adenovirus. **B** Sarcomere organization stained with phalloidin-TRITC conjugate; bar = 20 µm. Blue represent nucleus. Red represent F-actin. **C** Cell surface was calculated. *** *p* < 0.001. n = 3 experiments per group. **D** The mRNA levels of ANP and BNP were analyzed by qRT-PCR. The results were normalized to GAPDH. * *p* < 0.05. ** *p* < 0.01. n = 3 experiments per group. **E − G**, Knockdown of FOXO3a induced hypertrophic responses. Cardiomyocytes were infected with FOXO3a siRNA or FOXO3a scramble. **E** Sarcomere organization stained with phalloidin-TRITC conjugate; bar = 20 µm. Blue represent nucleus. Red represent F-actin. **F** Cell surface was calculated. * *p* < 0.05. n = 3 experiments per group. **G** The mRNA levels of ANP and BNP were analyzed by qRT-PCR. The results were normalized to GAPDH. * *p* < 0.05. ** *p* < 0.01. n = 3 experiments per group

Next, we explored the role of FOXO3a in cardiac hypertrophy. FOXO3a expression levels were analyzed in cardiomyocytes exposed to Ang II. Total FOXO3a levels were not altered, but phosphorylated FOXO3a increased upon Ang II treatment in a time-dependent manner (Fig. 6A), which indicated that Ang II induced loss of FOXO3a transcriptional activity. Enforced expression of FOXO3a attenuated Ang II-induced hypertrophy, and also significantly reduced the sarcomere organization, cell surface, and levels of hypertrophic marker ANP and BNP (Fig. 6B − D). In addition, cardiomyocytes with downregulated FOXO3a exhibited hypertrophic responses with increased sarcomere organization, increased cell surface and increased hypertrophic marker ANP and BNP in the absence of Ang II (Fig. 6E − G). Thus, it was concluded that FOXO3a inhibited Ang II-induced cardiac hypertrophy.

### FOXO3a regulates mitophagy in response to hypertrophy

**Fig. 7:**
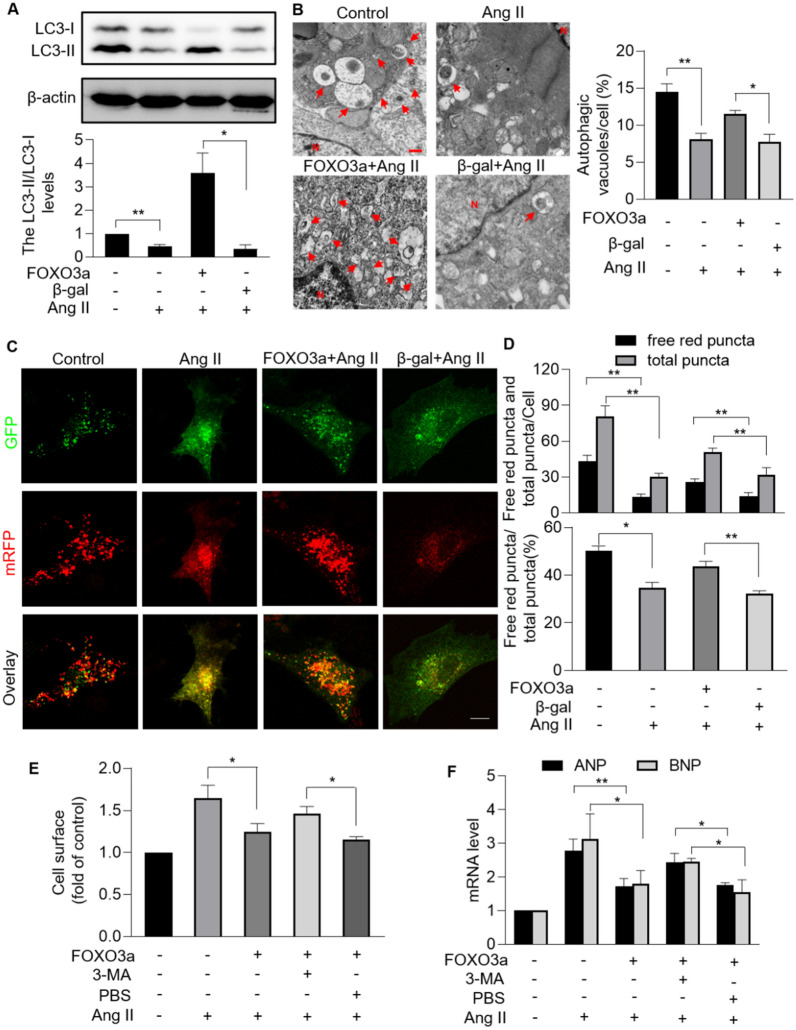
FOXO3a regulated cardiomyocyte mitophagy under hypertrophic stress. **A**, Enforced expression of FOXO3a rescued Ang II-induced decreased LC3II/LC3I. Cardiomyocytes were exposed to Ang II after infected with FOXO3a adenovirus or β-gal adenovirus. Immunoblotting was performed to detect protein levels of LC3I and LC3II (top). The ratio of LC3II/LC3I was calculated (bottom). * *p* < 0.05. ** *p* < 0.01. n = 3 experiments per group. **B**, FOXO3a restored autophagic vacuoles in hypertrophic model. Autophagic vacuoles were visualized in cardiomyocytes infected with FOXO3a adenovirus or β-gal (left; bar = 500 nm). Quantitation of autophagic vacuoles were shown in (right). * *p* < 0.05. ** *p* < 0.01. n = 3 experiments per group. **C** and **D**, Overexpression of FOXO3a attenuated Ang II-induced mitophagy flux defects. LC3 adenovirus tandem-labeled green fluorescent protein (GFP)-monomeric red fluorescent protein (mRFP) (GFP-mRFP-LC3) was used to indicate mitophagy flux. GFP-mRFP-LC3 was expressed and detected in 24 h after transfection in cardiomyocytes with overexpression or FOXO3a or not. GFP-LC3 (green puncta), mRFP (red puncta, representative of autolysosomes) and overlay (yellow puncta, representative of autophagosomes) (C; bar = 10 µm). Quantification results were shown in **D**. * *p* < 0.05. ** *p* < 0.01. n = 3 experiments per group. **E** and **F**, FOXO3a regulated cardiac hypertrophy through targeting mitophagy. After being infected with FOXO3a adenovirus, cardiomyocytes were treated with 3-Methyladenine (3-MA) or PBS, and then exposed to Ang II. (E) Cell surface area indicated by phalloidin-TRITC conjugate stained F-actin were calculated. *p < 0.05. n = 3 experiments per group. **F** The mRNA levels of ANP and BNP were detected. * *p* < 0.05. ** *p* < 0.01. n = 3 experiments per group

FOXO3a is deeply implicated in diverse cellular function including proliferation, differentiation, cell death, and mitochondrial fission. However, there is no substantial evidence that FOXO3a regulates mitophagy [30–33]. Therefore, we explored whether FOXO3a regulated mitophagy in hypertrophic cellular model. Ang II-induced decreased ratio of LC3-II/LC3-I were reversed by enforced expression of FOXO3a (Fig. 7A). More GFP-LC3II puncta co-localized with mitochondria were observed upon overexpression of FOXO3a than that of negative control group (Additional file 1: Fig. S1). Under hypertrophic stress, more mitophagic vacuoles accumulation was observed in cardiomyocytes overexpressing FOXO3a than that in cells overexpressed the negative control (Fig. 7B and Additional file 2: Fig. S2B). In addition, the effect of FOXO3a on mitophagic flux was analyzed. Overexpression of FOXO3a attenuated Ang II-induced mitophagic flux interruption, as demonstrated by increased ratio of free red puncta/total puncta (Fig. 7C, D). We then explored whether FOXO3a exerted an anti-hypertrophy function through promoting mitophagy. Enforced expression of FOXO3a reversed Ang II-induced hypertrophic responses including significantly increased cell surface and increased levels of hypertrophic markers ANP and BNP, which was counteracted by autophagy inhibitor 3-MA (Fig. 7E, F). In conclusion, FOXO3a promoted mitophagy process which was required for its hypertrophy-inhibiting effect.

### FOXO3a-dependent PARKIN mediates inhibition of hypertrophy through mitophagic pathway

**Fig. 8:**
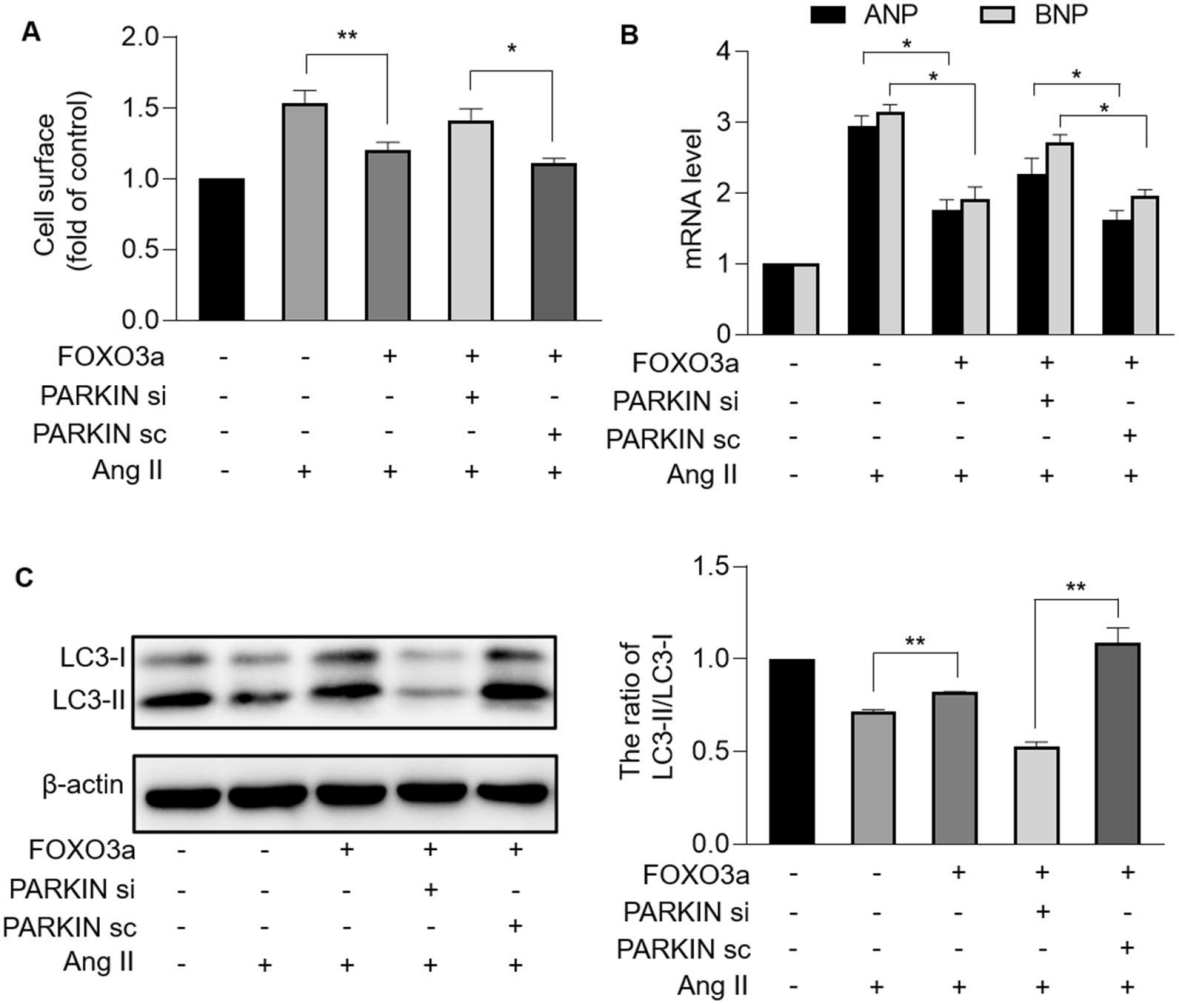
FOXO3a and PARKIN formed regulatory axis of mitophagy and hypertrophy. **A** and **B**, FOXO3a inhibited cardiomyocyte hypertrophy through targeting PARKIN. After being infected with FOXO3a adenovirus, cardiomyocytes were infected with PARKIN siRNA or PARKIN scramble, and then treated with Ang II. **A** Cell surface area indicated by phalloidin-TRITC conjugate stained F-actin were calculated. * *p* < 0.05. ** *p* < 0.01. n = 3 experiments per group. **B** The mRNA levels of ANP and BNP were detected. * *p* < 0.05. n = 3 experiments per group. **C**, FOXO3a regulated mitophagy through targeting PARKIN. After being infected with FOXO3a adenovirus, cardiomyocytes were infected with PARKIN siRNA or PARKIN scramble, and then treated with Ang II. Immunoblot were performed to detect protein levels of LC3I and LC3II. The ratio of LC3II/LC3I was calculated. ** *p* < 0.01. n = 3 experiments per group

We further investigated whether PARKIN was the target of FOXO3a in mitophagic and hypertrophic signaling pathways. Enforced expression of FOXO3a reversed the increase in cell surface and levels of hypertrophic marker ANP and BNP induced by Ang II, which was inhibited by knockdown of PARKIN (Fig. 8A, B). Cardiomyocytes with upregulated FOXO3a inhibited Ang II-induced mitophagy defect, which was counteracted by knockdown of PARKIN, as confirmed by decreased LC3-II/LC3-I ratio (Fig. 8C). Taken together, FOXO3a and PARKIN constituted a regulatory axis that functioned in cardiac mitophagy and hypertrophy.

## Discussion

Maladaptive cardiac hypertrophy eventually progresses to heart failure that is one of the top causes of death worldwide. It is necessary to discover the critical regulators and potential therapeutic targets in the pathogenesis of maladaptive cardiac hypertrophy and heart failure. Our present work identified PARKIN to be an anti-hypertrophy regulator. PARKIN could inhibit Ang II-induced cardiomyocyte hypertrophy. *Parkin* transgenic mice exhibited attenuated hypertrophic responses and cardiac remodeling, and improved cardiac function upon hypertrophic stress. In further exploring the underlying mechanisms, we found that mitophagy was impaired in response to Ang II, and PARKIN alleviated hypertrophy through restoring mitophagy. Moreover, we demonstrated that PARKIN was transcriptionally upregulated by FOXO3a. FOXO3a promoted mitophagy through activating PARKIN, which contributed to the anti-hypertrophic function of FOXO3a. Briefly, our results provide new insights into understanding the pathogenesis of cardiac hypertrophy and identify a novel anti-hypertrophic molecule.

*Parkin* was first identified in Parkinson’s disease (PD) and its loss-of-function mutation accounts much for the pathogenesis of PD. PARKIN has since been found to regulate other neurodegeneration diseases such as Alzheimer’s disease (AD) and Huntington’s disease (HD). Mitochondrial anomalies as well as impaired mitophagy have been observed in AD and HD. Enforced expression of PARKIN improves mitochondrial integrity and exerts a neuroprotection effect in these diseases [40, 41]. Beyond the role in nervous systems, PARKIN has been demonstrated to be a tumor suppressor through regulating a variety of cellular processes implicated in tumorigenesis, including cell cycle, cell proliferation, apoptosis, necroptosis, mitophagy, and metabolism [42]. Recent advances have implicated PARKIN in cardiac physiological and pathological processes. PARKIN is essential for heart development and its defect leads to dysplasia and premature death. Mice with cardiomyocyte-specific deletion of *Parkin* developed perinatal cardiomyopathy and premature death due to defective cardiac metabolic maturation [27]. Dilated cardiomyopathy was induced by knockout of *Parkin* in *Drosophila melanogaster* [43, 44]. In the mature hearts, PARKIN regulates ischemia/reperfusion injury, diabetic cardiomyopathy, and heart failure, but the role of PARKIN in pathological cardiac hypertrophy remains unknown [12, 19, 27–29]. In this study, we first substantially demonstrated that PARKIN negatively regulate cardiac hypertrophy and revealed the underlying mechanisms in *Parkin* transgenic mice and *Parkin*-overexpressing and -knockdown cardiomyocytes. We have already constructed *Parkin* knockout mice, but a sufficient number of mice for experiments has not yet been obtained so far due to *Parkin* deletion resulted in serious premature death and a very low survival rate. We will investigate the effect of Parkin deletion on hearts, especially whether Parkin deletion induced hypertrophy, using *Parkin* knockout mice in the future.

Cardiac hypertrophy is regulated by multiple pathways such as NFAT, AKT/mTOR, Ca^2^+/CaMKII, and cGMP-PKG [2–5]. These hypertrophic signaling mechanisms function generally through targeting a variety of processes including maladaptive gene expression and protein synthesis, cell growth, cell death, fibrosis, dysregulation of Ca^2^+-handling proteins, metabolic reprogramming, reactivation of fetal gene expression, and mitochondrial dysfunction [1]. Albeit mitochondrial morphological and functional defects contribute to pathological enlargement of hearts, the association between mitophagy (the mitochondrial quality control system) and hypertrophy regulation is still elusive and related studies are limited. Sadoshima et al. found that mitophagy and autophagy alter in two distinct manners in response to pressure overload. Injection of non-mitophagy-specific autophagy inducer rescues mitophagy, attenuates mitochondrial dysfunction, and improves cardiac function in failing hearts [45]. Our results indicated that mitophagy were impaired in both cardiomyocytes and hearts exposed to Ang II, which could be reversed by overexpression of mitophagy inducer PARKIN. PARKIN-dependent mitophagy attenuated cardiac hypertrophy and remodeling, and improved cardiac function, playing a cardio-protection role. Research has revealed that mitophagy could also be regulated independently of PARKIN. Mitophagy receptors on the OMM, such as Bcl2/adenovirus E1B 19 kDa protein-interacting protein 3 (BNIP3), Nip3-like protein X (NIX), Fun14 Domain containing 1 (FUNDC1), and Bcl-2-like protein 13 (BCL2-L-13) have been demonstrated to directly bind to LC3 and target mitochondria removal [46, 47]. Thus, whether PARKIN-independent mitophagy participates in cardiac hypertrophy is an interesting question for future exploration.

PARKIN is an E3 ubiquitin ligase and generally functions in a ubiquitin–proteasome dependent way. Hitherto, a dozen OMM substrates of PARKIN in mediating mitophagy such as MFN1/2, MIRO, and VDAC1/2/3, and the fundamental cascades have been well elucidated [22–25], yet its upstream regulatory mechanisms are not well understood. PINK1 is a widely recognized regulator of PARKIN in mitophagic signaling pathways. Upon phosphorylated by PINK1, PARKIN is recruited to the outer membrane of the damaged mitochondria where PARKIN extensively catalyzes ubiquitination of OMM substrates [22, 25]. During above processes, another E3 ubiquitin ligase MDM2 accumulates to OMM and enhances enzymatic activity of PARKIN, whereas cytosolic p53 hinders PARKIN from translocation to damaged mitochondria and PICK1 suppresses E3 ligase activity of PARKIN [48–50]. Our results identified FOXO3a as a novel regulator of PARKIN, in which FOXO3a promotes *Parkin* transcription by binding to its promoter region, and activates PARKIN-dependent mitophagy.

A growing body of evidence has shown that FOXO3a participates in the pathogenesis of cardiac disorders through regulating diverse cellular processes such as necroptosis, apoptosis, calcium homeostasis, hypertrophy, and mitochondrial fission [30–32, 34–36]. Recent research has suggested that FOXO3a is implicated in the regulation of mitophagy, as evidenced by its effect on the expression of mitophagy-related protein BNIP3, and co-inactivation and co-localization with PARKIN [17, 37, 38]. However, there is no direct evidence to show that FOXO3a can regulate mitophagy. Our present work substantially demonstrated the role of FOXO3a in mitophagic program. Overexpression of FOXO3a promoted formation of mitophagosomes, transformation of LC3-I to LC3-II and autophagic flux. FOXO family contains four members including FOXO1, FOXO3a, FOXO4 and FOXO6. A recent study has shown that FOXO1 promotes mitophagy through transcriptionally targeting PINK1 in podocytes [51]. It would be interesting to explore whether other members of FOXO family participate in cardiac mitophagy program. We have demonstrated that FOXO3a activated *Parkin* transcription by directly binding to the consensus sequence located at the promoter region of *Parkin*. As FOXO3a potential binding sites could recognized by other FOXO family members including FOXO1 [52, 53], we wonder whether Parkin expression could be regulated by FOXO1. It was showed that neither overexpression nor knockdown of FOXO1 affected the protein expression levels of PARKIN in H9c2 cells (Additional file 3: Figure S3). We will investigate the interaction between *Parkin* and other FOXO family members in future.FOXO3a has been documented to function through a transcriptional mechanism and its transcriptional activity is controlled by post-translational modification such as phosphorylation. The dephosphorylated FOXO3a accumulates in nucleus and regulates gene transcription, while the phosphorylated FOXO3a are exported to cytoplasm and loses transcriptional activity [54]. Our results showed that phosphorylated FOXO3a increased upon Ang II treatment, which indicated that Ang II decreased FOXO3a transcriptional activity. Enforced expression of FOXO3a rescued its activity repression by Ang II and enhanced transcription of the nuclear gene *Parkin*. FOXO3a participated in mitophagic program through upregulating PARKIN at the transcriptional level. Intriguingly, a recent study has revealed that a shorter FOXO3a isoform locates in mitochondria of both normal and cancer cells and regulates mitochondrial gene transcription and mitochondria homeostasis [55]. Moreover, it was reported that FOXO3a seemly co-localizes with mitochondrial PINK1 and PARKIN [38], which probably predicts that FOXO3a shuttles into mitochondria and more directly regulates mitophagy. Thus, future studies are required to elucidate whether FOXO3a regulates mitophagy in other ways occurring in mitochondria such as affecting ubiquitination of OMM substrates or recruiting autophagy adaptors.

## Conclusions

In summary, this work reveals a highlighted connection between PARKIN-dependent mitophagy and pressure overload-induced cardiac hypertrophy. Mitophagy is impaired in cardiomyocytes treated with Ang II. PARKIN inhibits cardiac hypertrophy and remodeling through restoring mitophagy. FOXO3a transcriptionally upregulates PARKIN and regulates mitophagy, which contributes to the cardio-protective effect of FOXO3a. Our results revealed a novel cardiac hypertrophic regulating model composed of FOXO3a, PARKIN and mitophagy, which may lead to future studies to explore not only the implications of this model in hypertrophy, but also the application as potential therapeutic targets and strategies for cardiac hypertrophy and heart failure.

## Methods

### Generation of transgenic mice with cardiac-specific overexpression of *Parkin*

Cardiac-specific *Parkin* transgenic mice were generated as previously described [19]. cDNAs containing murine *Parkin* were obtained from mice hearts through RT-PCR and cloned into pαMHC-clone26 vector under the control of the α-myosin heavy chain (α-MHC) promoter. After linearization and purification, the recombined vectors were microinjected into 0.5 dpc zygote. The genotyping of *Parkin* transgenic mice was identified by PCR. The PCR primers include: forward primer in the α-MHC promoter, 5′- AGTGGTGGTGTAGGAAAGTC-3′, and the reverse primer in the *Parkin* DNA, 5′- TGCTTCTGAATCCCTCTTAC-3′.

## Cell culture and treatment

Neonatal rat cardiomyocytes were isolated from 1 to 2-day-old SD rats as previously described [19]. Hearts of the dissected rats were removed, thoroughly cleaned and minced in iced PBS solution. Then the tissues were dispersed and incubated at 37 °C in PBS solution containing 1.2 mg/ml pancreatin and 0.14 mg/ml collagenase type II (Worthington) for several times. After removing non-cardiomyocytes by differential adhension method, cardiomyocytes were collected and plated at poly-L-lysin coated different culture dishes in Dulbecco’s modified Eagle medium/F-12 (Hyclone) supplemented with 5% heat-inactivated fetal bovine serum, 100 U/ml penicillin, 100 µg/ml streptomycin, and 0.1 mM bromodeoxyuridine. H9c2 cells and HEK293 cells were cultured in Dulbecco’s modified Eagle medium (Hyclone) supplemented with 10% fetal bovine serum, 100 U/ml penicillin, 0.1 mg/ml streptomycin, and 110 mg/ml sodium pyruvate in a humidified atmosphere containing 5% CO_2_ at 37 °C. Neonatal rat cardiomyocytes and H9c2 were treated with 1 µM Angiotensin II (Merck millipore) for 24 h except as otherwise indicated.

## Determination of sarcomere organization and cell surface areas

Cardiomyocytes were fixed in 4% paraformaldehyde in PBS for 10 min at room temperature and then washed three times in PBS for 5 min. After dehydration with acetone for 3 min and treatment with 0.1% Triton X-100 for 20 min, cardiomyocytes were stained with a 100 nM fluorescent Phalloidin-TRITC conjugate (Sigma) for 30 min at room temperature. Then the cells were mounted with fluorescent mounting medium with DAPI and visualized by a laser confocal microscopy (Olympus).

## Quantitative reverse transcription-PCR (qRT-PCR)

qRT-PCR for ANP and BNP mRNA was performed on an Applied Biosystems ABI Prism QuantStudio 3. Total RNA was extracted by Trizol reagent (Invitrogen). After DNAse I incubation, the RNAs were reverse transcribed into cDNAs with oligo-d(T) and random primers using a reverse transcriptase kit (Takara). The expression levels of mRNA were detected by real time PCR using a SYBR® Premix Ex Taq™ (Takara). The sequences of rat ANP primers were: forward, 5'- GGCTCCTTCTCCATCC-3'; reverse, 5'- TGTTATCTTCGGTAC-3'. The sequences of mouse ANP primers were: forward, 5’-TAGGAGACAGTGACGGACAA-3’; reverse, 5’-GAAGAAGCCCAGGGTGAT-3’. The sequences of rat BNP primers were: forward, 5'- GACGGGCTGAGGGT-3'; reverse, 5'- ACTGTGGCAAGTGTGCTG -3'. The results were standardized to those of GAPDH. The sequences of rat GAPDH primers were: forward, 5'- TGGAGTCTACTGGCGTCTT -3'; reverse, 5'- TGTCATATTTCTCGTGGTTCA-3'. The sequences of mouse GAPDH primers were: forward, 5’-TGTGTCCGTCGTGGATCTGA-3’; reverse, 5’-CCTGCTTCACCACCTTCTTGA-3’.

## Detection of GFP-mRFP-LC3 puncta

Autophagic flux was detected using LC3 adenovirus tandem-labeled green fluorescent protein (GFP)-monomeric red fluorescent protein (mRFP) (GFP-mRFP-LC3). The green fluorescence is weakened when autophagosome/mitophagosome fuse to lysosome, as GFP is normally deprotonated in acidic lysosomes. Thus, yellow puncta indicated autophagosome/mitophagosome and free red puncta indicated autolysosomes.

Cells were infected with GFP-mRFP-LC3 adenovirus. GFP and mRFP fluorescence was imaged using a laser scanning confocal microscope (Olympus). The number of red, green and yellow puncta cells were quantified.

## Adenoviral constructs and infection

*Parkin* and *Foxo3a* adenoviral constructs were prepared as previously described [19]. In brief, rat *Parkin* and *Foxo3a* cDNAs were obtained from rat hearts using RT-PCR, and then cloned into the Adeno-X™ Expression System (Clontech) according to the manufacturer’s instructions. Adenoviruses were produced and amplified in HEK293 cells. The adenovirus containing β-galactosidase (β-gal) was constructed as control. Cardiomyocytes were infected with adenoviruses at 150 multiplicities of infection (moi) in the absence of fetal bovine serum for 2 h.

## siRNA construction of PARKIN and FOXO3a

The sense sequence of PARKIN siRNA was 5’-TTCCAAACCGGATGAGTGG-3’, and the antisense sequence was 5’-CCACTCATCCGGTTTGGAA-3’. The scramble PARKIN siRNA sense sequence was 5’-GCTCATCGAGCTAGTAGAG-3’, and the scramble antisense sequence was 5’-CTCTACTAGCTCGATGAGC-3’.

The FOXO3a siRNA sense sequence was 5’-GAGCTCTTGGTGGATCATC-3’; the antisense sequence was 5’-GATGATCCACCAAGAGCTC-3’. The scramble FOXO3a siRNA sense sequence was 5’-GGCGTAGTCGTAGTTCTCA-3’; the scramble antisense sequence was 5’-TGAGAACTACGACTACGCC-3’. These siRNAs were cloned into the pSilencer 1.0-CMV System (Ambion) according to the manufacturer’s instructions. Adenoviruses were produced and amplified in HEK293 cells.

## Luciferase reporter construct and transfection of *Parkin* promoter sensor reporter

*Parkin* promoter (total 1662 bp from the 1596 bp upstream of *Parkin* to the 26th bp of *Parkin,* containing Foxo3a potential binding site) and *Parkin* mutated promoter (mutated the Foxo3a potential binding sites) were synthesized from Sangon Biotech. Then the DNA fragments were cloned into pGL4 vector, downstream of the coding region of luciferase gene.

## Chromatin immunoprecipitation (ChIP) assay

ChIP was performed using ChIP kit (Abcam, ab500) according to the manufacturer’s instructions. In brief, cells were crosslinked in formalin for 10 min at room temperature, which was then quenched with 125 mM glycine for 5 min. Cells were washed thrice with iced PBS and lysed in ChIP lysis buffer at 4 ℃ for 10 min. Then cell lysates were sonicated into chromatin fragments with a length of 200 − 1000 bp at 4 ℃. Agarose gel electrophoresis was performed to detect the length of fragments. Protein A was blocked with single chain herring sperm DNA and BSA at room temperature for 30 min. The chromatin fragments were incubated with the blocked protein A overnight at 4 ℃. The DNA was eluted and purified from the immunoprecipitation. The purified DNA was analyzed using PCR with the following primers that encompass FOXO3a binding sites of *Parkin* promoter. The sequences of PCR primers were: forward, 5’- CACTCAGTAGACGACCTT-3’; reverse, 5’- GTCCCTAATAATACAAGC-3’.

## Immunoblotting

Immunoblotting was carried out as we previously described [56]. In brief, cells were lysed at 4 ℃ for 1 h using RIPA lysis buffer (Solarbio, Beijing, China) containing 0.1 mM phenylmethanesulfonyl fluoride (PMSF) and a protease inhibitor (Roche). After centrifugation, the total protein was obtained and subjected to 10 − 12% SDS-PAGE according to the molecular weight of protein. Then the protein was transferred to nitrocellulose membranes. Blots were probed using antibodies including anti-PARKIN (Abcam, ab15954), anti-LC3A/B (Cell Signaling Technology, #4108), anti-FOXO3a (Abcam, ab17026), and anti-phospho-FOXO3a (Abcam, ab47285) antibodies. The antigen–antibody complexes were visualized by ECL western blotting substrate (Boster) on a Bio-Rad ChemiDoc system. Western blot bands were quantified using the ImageJ software.

## Transmission electron microscopy (TEM)

Conventional TEM was performed as previously described [19]. Briefly, cells were successively treated with 2.5% glutaraldehyde, 1% osmium tetraoxide, and a graded series of ethanol concentrations. Then the samples were embedded in Embed812 resin and underwent ultramicrotomy. After being mounted on copper grids, the samples were stained with uranyl acetate and lead citrate, and visualized with a FEI Tecnai spirit transmission electron microscope.

## Animal experiments

All animal experiments were performed using protocols [57] that adhered to the standards of the National Institute of Health Guide for the Care and Use of Laboratory Animals, under authorized protocols approved by the Animal Ethics Committee of Shanxi Medical University (Taiyuan, China). Adult C57BL6/J mice (Beijing Vital River Laboratory Animal Technology Co., Ltd.) at 8 weeks of age were used in the study. Wild type mice (C57BL6/J) and transgenic mice at 8 weeks of age were infused with Ang II (Merk Millipore, 0.6 mg/kg/day dissolved in 0.9% NaCl) for 2 weeks. The infusions were executed with implanted osmotic minipumps (Alzet model 1002, Alza Corp.) according to the manufacturer’s instructions. Briefly, after mice were anesthetized, a mid-scapular incision was made on their back. A hemostat was inserted into the incision, and by opening and closing the jaws of the hemostat, the subcutaneous tissue is spread to create a pocket for the pump on the left back of mice. A pump filled with Ang II was then inserted into the pocket and the wound was closed with wound sutures.

## Echocardiography and histological staining

Echocardiography was carried out as we described previously [19]. In brief, two weeks after Ang II infusions, transthoracic echocardiography was performed using a Vevo 770 high-resolution system (Visualsonics) equipped with a 40 MHz RMV 704 scanhead. End-systolic interventricular septum thickness (IVSs) was measured. After in vivo evaluation of cardiac function, mice were euthanized, and hearts were harvested and stained with hematoxyline-eosin (HE), Masson trichrome, and TRITC-conjugated wheat germ agglutinin (WGA). HE staining and standard Masson trichrome staining (Solarbio) was used to assess myocardial fibrosis induced by Ang II according to the manufacturer’s instructions. TRITC-WGA (Sigma) staining was performed to determine cross-sectional area according to the manufacturer’s instructions.

## Statistical analysis

Data were expressed as mean ± standard deviation (SD) of at least three independent experiments. Student *t* test was used for two groups. One-way analysis of variance was used for multiple group comparisons. A value of *p* < 0.05 was considered statistically significant.

## Supplementary Information

**Additional file 1: Figure S1.** PARKIN and FOXO3a regulated mitophagy in hypertrophyic model. **A.** After transfected with GFP-LC3 vector, the cardiomyocytes were infected with PARKIN or FOXO3a adenovirus, and then treated with Ang II. GFP-LC3II puncta co-localized with mitochondria was analyzed followed by MitoTr. Green represent LC3. Red represent mitochondria. Blue represent nucleus. Bar = 20 µm.** B.** The GFP-LC3-puncta positive cells was calculated. n=3 experiments per group. * *p *<0.05.

**Additional file 2: Figure S2.** PARKIN and FOXO3a restored mitophagic vacuoles in hypertrophic model. Mitophagic vacuoles were visualized in cardiomyocytes infected with PARKIN or FOXO3a adenovirus (bar = 200 nm).

**Additional file 3: Figure S3.** FOXO1 dose not regulate PARKIN expression. Immunoblotting results showing the protein levels of PARKIN in cardiomyocytes infected with FOXO1 adenovirus or FOXO3a siRNA. n=3 experiments per group. ns: no significance.

## Data Availability

Data are available upon reasonable request by sending a message to the corresponding author.

## Abbreviations

NFAT: Nuclear factor of activated T cell
AKT: AMP-activated protein kinase
mTOR: Mammalian target of rapamycin
CaMK: Calmodulin-dependent protein kinase
cGMP: Cyclic guanosine monophosphate
PKG: Protein kinase G
MAPK: Mitogen-activated protein kinase
RIPK3: Receptor-interacting serine/threonine-protein kinase 3
CYPD: Cyclophilin-D
MOM: Mitochondrial outer membrane
PINK1: PTEN-induced putative kinase protein-1
MFN: Mitofusion
SQSTM1: Sequestosome 1
OPTN: Optineurin
PD: Parkinson’s disease
FOXO3a: Forkhead box O3a
Ang II: Angiotensin II
BNIP3: Bcl-2 E1B 19-KDa interacting protein 3
MHC: Myosin heavy-chain
ANP: Atrial natriuretic peptide
LC3: Light chain 3
TEM: Transmission electron microscopy
GFP: Green fluorescent protein
mRFP: Monomeric red fluorescent protein
OMM: Outer mitochondrial membrane
3-MA: 3-Methyladenine
PI3K: Phosphatidylinositol 3-kinase
ChIP: Chromatin immunoprecipitation
AD: Alzheimer’s disease
HD: Huntington’s disease
NIX: Nip3-like protein X
FUNDC1: Fun14 Domain containing 1
BCL2-L-13: Bcl-2-like protein 13
PMSF: Phenylmethanesulfonyl fluoride
IVSs: Interventricular septum thickness
HE: Hematoxyline-eosin
WGA: Wheat germ agglutinin
SD: Standard deviation
