# Supplementary figures and images for: FOXO3a-dependent PARKIN negatively regulates cardiac hypertrophy by restoring mitophagy
Source: Cell Biosci. 2022 Dec 19;12:204. doi: 10.1186/s13578-022-00935-y (PMC9764573; doi:10.1186/s13578-022-00935-y)

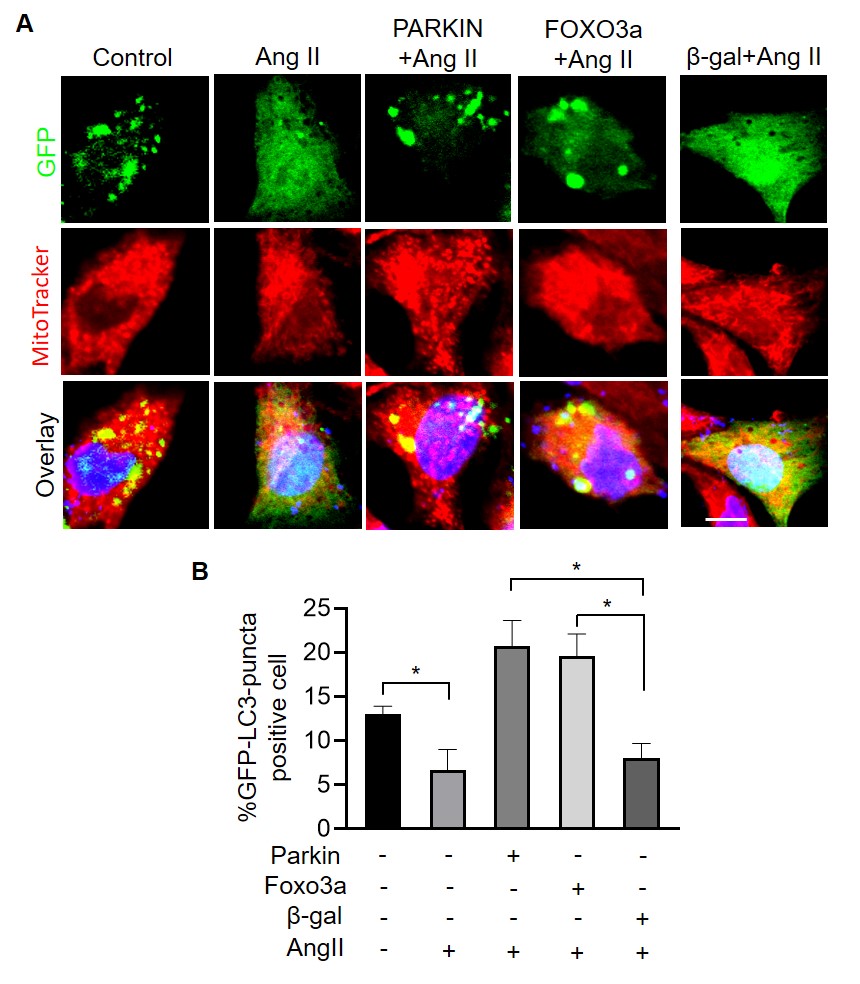

Supplement: Supplementary file 1 — Additional file 1: Figure S1. PARKIN and FOXO3a regulated mitophagy in hypertrophyic model. A. After transfected with GFP-LC3 vector, the cardiomyocytes were infected with PARKIN or FOXO3a adenovirus, and then treated with Ang II. GFP-LC3II puncta co-localized with mitochondria was analyzed followed by MitoTr. Green represent LC3. Red represent mitochondria. Blue represent nucleus. Bar = 20 µm. B. The GFP-LC3-puncta positive cells was calculated. n=3 experiments per group. * p <0.05. [file 13578_2022_935_MOESM1_ESM.jpg]

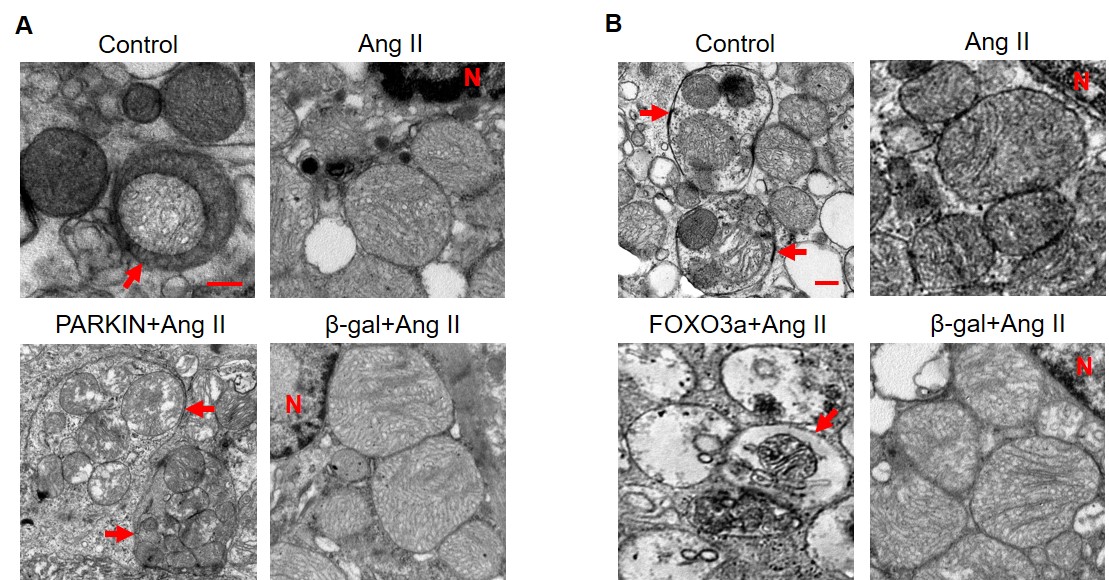

Supplement: Supplementary file 2 — Additional file 2: Figure S2. PARKIN and FOXO3a restored mitophagic vacuoles in hypertrophic model. Mitophagic vacuoles were visualized in cardiomyocytes infected with PARKIN or FOXO3a adenovirus (bar = 200 nm). [file 13578_2022_935_MOESM2_ESM.jpg]

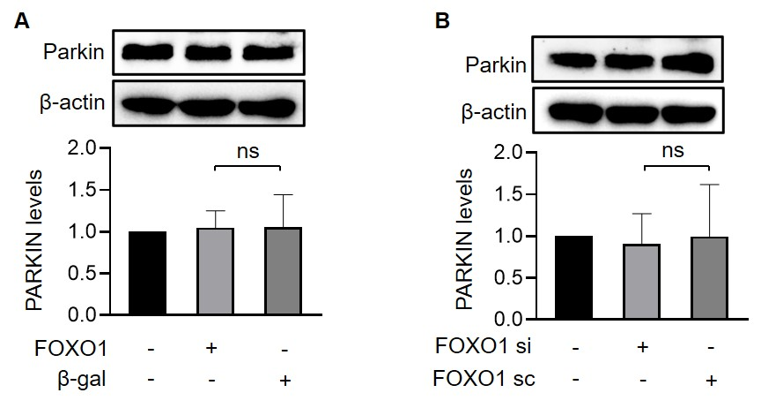

Supplement: Supplementary file 3 — Additional file 3: Figure S3. FOXO1 dose not regulate PARKIN expression. Immunoblotting results showing the protein levels of PARKIN in cardiomyocytes infected with FOXO1 adenovirus or FOXO3a siRNA. n=3 experiments per group. ns: no significance. [file 13578_2022_935_MOESM3_ESM.jpg]
